# Genome-wide characterization of the *CIPK* gene family in mung bean and functional validation of *VrCIPK5* in drought stress response

**DOI:** 10.3389/fpls.2026.1866081

**Published:** 2026-07-20

**Authors:** Lili Yin, Jiaqi Liao, Jie Gao, Yonglin Xiao, Wenye Hu, Ruigang Wu, Xiaoliang Chen

**Affiliations:** 1 College of Agronomy and Life Sciences, Shanxi Datong University, Datong, China; 2 School of Medicine, Shanxi Datong University, Datong, China; 3 School of Landscape and Ecological Engineering, Hebei University of Engineering, Handan, China; 4 Institute of Respiratory and Occupational Diseases, Shanxi Datong University, Datong, China

**Keywords:** *CIPK* gene family, drought stress, gene expression, gene function, mung bean

## Abstract

Calcineurin B-like protein-interacting protein kinases (CIPKs) act as core regulators in plant calcium (Ca^2+^) signaling pathways and mediate abiotic stress adaptation. Mung bean (*Vigna radiata* L.) is an economically important legume that is widely grown in arid and semi-arid regions; however, drought stress significantly reduces its yield and quality. Despite this, genome-wide identification and functional analysis of the *CIPK* gene family have not been reported in mung bean, limiting our understanding of drought-resistance mechanisms and stress-tolerant variety breeding. In this study, 23 *VrCIPK* genes were identified from the mung bean genome, which were unevenly distributed across 6 chromosomes, with the remaining genes located on scaffolds. Phylogenetic analysis classified the *VrCIPK* family into five groups (A–E), characterized by high structural conservation of the N-terminal kinase and the C-terminal NAF/FISL regulatory domains. Gene Ontology annotation further indicated their conserved involvement in protein phosphorylation and calcium signal transduction. Collinearity analysis revealed that *CIPK* genes in mung bean and other species have relatively conserved evolutionary patterns. Segmental duplication contributed to *VrCIPK* gene family expansion, and most duplicated gene pairs underwent purifying selection during evolution. Gene structure and motif analyses showed that *VrCIPK* genes within the same group shared conserved structural features. Cis-acting element profiling revealed abundant hormone- and stress-responsive elements in *VrCIPK* promoters, indicating diverse transcriptional regulatory potential. Transcriptomics analysis and quantitative real-time PCR demonstrated that *VrCIPK5* was significantly induced under drought stress. The heterologous overexpression of *VrCIPK5* in tobacco conferred enhanced drought tolerance by promoting proline accumulation, increasing antioxidant enzyme (superoxide dismutase, peroxidase, and catalase) activities, accelerating stomatal closure, and upregulating downstream stress-responsive genes (*NtSOD*, *NtCAT*, *NtP5CS1*, *NtLEA5*, and *NtRD29A*), thus alleviating reactive oxygen species overaccumulation and membrane lipid peroxidation. Collectively, these findings fill the research gap in the genome-wide identification and functional analysis of the *CIPK* gene family in mung bean and provide key genetic resources and theoretical support for breeding drought-resistant mung bean varieties.

## 1 Introduction

Calcium ions (Ca^2+^) act as key secondary messengers in plants, regulating abiotic stress responses and development. Ca^2+^ signaling is a central mechanism by which plants adapt to environmental stressors (Ranty et al., 2016). Intracellular Ca^2+^ levels show rapid and specific fluctuations under drought, salinity, and cold stress. These Ca^2+^ signatures act as a “molecular code” for stress perception, supporting signal decoding and downstream responses (Kolukisaoglu et al., 2004; Luan, 2009). Calcineurin B-like proteins (CBLs) are plant-specific Ca^2+^ sensors that lack kinase activity and therefore cannot directly amplify signals. Upon Ca^2+^ binding, CBLs interact with CBL-interacting protein kinases (CIPKs), forming functional complexes that phosphorylate downstream targets and activate physiological and biochemical cascades that enhance plant stress tolerance (Kudla et al., 1999; Luan et al., 2002; Reddy and Reddy, 2004).

CIPKs belong to a family of plant-specific serine/threonine protein kinase family, and their highly conserved modular structure provides the core molecular basis for mediating signal transduction and achieving the precise regulation of kinase activity (Chaves-Sanjuan et al., 2014). All CIPKs proteins contain two functionally well-characterized core domains: an N-terminal kinase catalytic domain and a C-terminal regulatory domain. The N-terminal kinase domain contains conserved DFG and APE motifs; phosphorylation of serine (S), threonine (T), and tyrosine (Y) residues in the activation loop between these two motifs induces a conformational switch in CIPK from an autoinhibited state to an active state, providing the enzymatic basis for the phosphorylation of downstream target proteins (Guo et al., 2001). The core of the C-terminal regulatory domain is a 24-amino acid (aa) NAF/FISL motif, which has dual functions. Under normal conditions, it maintains CIPK in an autoinhibited state through an intramolecular interaction with the N-terminal kinase domain, masking the active center. Under stress conditions, it acts as an interaction interface to mediate specific binding between CIPK and CBL, relieving intramolecular inhibition and activating CIPK kinase activity (Albrecht et al., 2001; Hashimoto et al., 2012).

Previous studies have demonstrated that *CIPK* family members play pivotal roles in the responses of plants to abiotic stress. In *Arabidopsis thaliana*, the interaction between AtCIPK24 and AtCBL4 drives the phosphorylation and activation of Na^+^/H^+^ exchanger SOS1, which extrudes excess intracellular Na^+^, modulating salt tolerance (Yang et al., 2019). AtCBL1 interacts with AtCIPK1, maintaining cellular osmotic balance in *Arabidopsis* (Cheong et al., 2003), and AtCIPK23 interacts with CBL1/CBL9, regulating abscisic acid (ABA)-mediated drought stress responses (Mao et al., 2016). AtCIPK21 interacts with AtCBL2/AtCBL3, mediating responses to high salt, hyperosmotic, and cold stress (Chen et al., 2013). In *Cajanus cajan*, the interaction between CcCBL1 and CcCIPK14 enhances flavonoid biosynthesis, improving drought tolerance (Meng et al., 2021). In Manihot esculenta, MeCIPK23 interacts with MeWHY transcription factors, activating the expression of MeNCED1, increasing ABA biosynthesis and regulating drought resistance (Yan et al., 2020). The overexpression of *CIPK* genes alters the tolerance of plants to abiotic stress. For instance, the overexpression of *OsCIPK3* or *OsCIPK15* in rice enhances the responsiveness of plants to low temperature, drought, and salt stress (Xiang et al., 2007). Heterologous expression of wheat *TaCIPK2*, *TaCIPK23*, or *TaCIPK27* in *Arabidopsis* improves the drought tolerance of transgenic plants through ABA-mediated signaling pathways (Wang et al., 2018). The overexpression of maize *ZmCIPK8* in tobacco confers enhanced drought resistance (Tai et al., 2016). Transgenic tomato lines overexpressing *MdCIPK6* show significantly enhanced tolerance to low temperature, high salinity, and drought stress (Wang et al., 2012a). Collectively, these findings indicate that the *CIPK* gene family represents a valuable candidate gene resource pool for breeding stress-resistant crops.

Mung bean (*Vigna radiata* L.) is a nutrient-rich legume crop with high protein, moderate starch, and low fat content, and is widely used as both medicine and food. Its nitrogen-fixing ability gives it an important ecological value in crop rotation systems. However, mung bean yield and quality are frequently threatened by abiotic stressors. Moreover, traditional mung bean breeding is constrained by limited genetic variation in germplasm resources and low heritability of stress-tolerance traits, leading to low efficiency in the breeding of stress-tolerant varieties (Nair et al., 2019). Therefore, mining stress-resistance genes from mung bean itself provides high-quality homologous gene resources for genetic engineering and accelerates the development of stress-tolerant mung bean varieties. Although the *CIPK* gene family has been identified in plant species including *Arabidopsis thaliana* (Kolukisaoglu et al., 2004), rice (Kanwar et al., 2014), soybean (Zhu et al., 2016), tomato (Zhang et al., 2019), and potato (Ma et al., 2021), the systematic identification and functional characterization of the *CIPK* gene family in mung bean remain unreported in mung bean. In this study, 23 *VrCIPK* genes were identified in the mung bean genome, and their phylogenetic relationships, gene structures, and characteristics were systematically analyzed. The expression patterns of *VrCIPK* genes were detected under drought stress, and the drought tolerance of *VrCIPK5* was verified using transgenic tobacco. This study provides gene resources and a theoretical basis for the molecular breeding and genetic improvement of stress resistance in mung bean.

## 2 Materials and methods

### 2.1 Genome-wide identification of the *VrCIPK* gene family

Genome data and structural annotation information of mung bean (*Vigna radiata* L.) were downloaded from the Ensembl Plants Database (http://plants.ensembl.org/index.html). The reference genome assembly version used in this study was Vradiata_ver6. The CIPK sequences of *A. thaliana* were retrieved from the TAIR Database (https://www.arabidopsis.org/). HMM files corresponding to the protein kinase domain (PF00069) and NAF/FISL domain (PF03822) were obtained from the Pfam Database (http://pfam.xfam.org/), and the mung bean protein database was scanned using HMMER software (v3.0) (Finn et al., 2014). Furthermore, homologous alignment was performed across the whole genome of mung bean via the BLASTP program with an E-value threshold of ≤1e–5. Candidate sequences identified by the two methods were combined, and redundant sequences were removed. The combined non-redundant dataset contained a small subset of candidates that were uniquely detected by one of the two analytical methods. The resulting sequences were submitted to the SMART Database (http://smart.embl-heidelberg.de/) (Letunic et al., 2015), NCBI Conserved Domain Database (CDD, https://www.ncbi.nlm.nih.gov/cdd) and Pfam Database (http://pfam.xfam.org/) (Wilkins et al., 1999) for conserved domain validation. The simultaneous presence of conserved Pkinase and NAF domains was set as the defining criterion for candidate kinases to be classified into the *CIPK* gene family. The genes were named according to their chromosomal distribution order and their chromosomal distribution map was drawn using MapChart software. The molecular weight (MW), theoretical isoelectric point (pI) and amino acid (aa) sequence length of VrCIPK proteins were computed using the ExPASy Server (http://web.expasy.org/), and subcellular localization prediction was performed with the WoLF PSORT Server (https://wolfpsort.hgc.jp/).

### 2.2 Phylogenetic analysis of mung bean *CIPK* gene family

CIPK protein sequences from *V. radiata*, *A. thaliana*, *Oryza sativa*, and *Glycine max* were aligned using ClustalW (Thompson et al., 1994). An unrooted phylogenetic tree was constructed in MEGA 7.0 using the Neighbor-Joining (NJ) method with 1000 bootstrap replicates (Kumar et al., 2016).

### 2.3 Synteny analysis of the *CIPK* gene family

To explore interspecific evolutionary relationships of the *CIPK* family between mung bean and *Arabidopsis thaliana*, *Oryza sativa*, *Glycine max*, chromosome-level genome sequences and GFF3 annotations of the four species were downloaded. Whole-genome BLAST alignment combined with gene chromosomal locations was used to identify syntenic blocks via MCScanX (Wang et al., 2012b). CIPK-containing homologous pairs were screened, and syntenic relationships between mung bean and the other three species were visualized. For intraspecific analysis in mung bean, MCScanX was used to detect intragenomic syntenic blocks and *CIPK* duplicated gene pairs. A synteny map was constructed using Circos version 0.69-9 (Krzywinski et al., 2009). The non-synonymous substitution rate (Ka) and synonymous substitution rate (Ks) values of duplicated pairs were calculated with Calculator 2.0 (Wang et al., 2010), and selection pressure was determined by the Ka/Ks ratio.

### 2.4 Analysis of gene structure and conserved motifs of *VrCIPK* genes

Gene structure analysis was conducted via the GSDS version 2.0 (http://gsds.gao-lab.org/) (Guo et al., 2007), and schematic diagrams of gene structures including exons, introns and untranslated regions (UTRs) were obtained. Conserved motifs were analyzed using the MEME version 5.4.1 (http://meme-suite.org/tools/meme).

### 2.5 Gene ontology enrichment analysis

Gene Ontology (GO) annotation information of mung bean was downloaded using the BioMart tool on the Ensembl Plants platform. We retrieved GO terms corresponding to three main functional categories, including Biological Process (BP), Molecular Function (MF), and Cellular Component (CC), for all 23 identified *VrCIPK* genes. GO enrichment analysis for the *VrCIPK* family was performed, and a chord diagram was generated to present the results using TBtools-II version 2.420.

### 2.6 Analysis of cis-acting elements in the promoters of *VrCIPK* genes

The 1500 bp genomic sequence upstream of the translation start codon (ATG) of each *VrCIPK* gene was extracted from the mung bean reference genome and defined as the core promoter region. The above sequences were then submitted to the PlantCARE online analysis tool (https://bioinformatics.psb.ugent.be/webtools/plantcare/html/) to identify cis-acting elements associated with hormone and stress responses on the promoters of *VrCIPK* genes (Lescot et al., 2002).

### 2.7 Expression of *VrCIPK* genes under drought stress

The analysis of the expression patterns of *VrCIPK* genes under drought stress was based on the NCBI SRA Database (BioProject: PRJNA764584). Non-expressed genes with transcripts per kilobase of exon model per million mapped reads (TPM) value of 0 in all biological replicates of both treatment and control groups were excluded. To avoid zero values and stabilize variance during log transformation, 1 was added to all TPM values prior to log_2_ transformation. The TPM values were normalized across samples to ensure comparability, and log_2_(fold change) = log_2_(treatment TPM/control TPM) was calculated for differential expression analysis. Genes with FDR < 0.05 and |log_2_FC| > 1 were considered significantly differentially expressed. The heatmap was generated with TBtools-II version 2.420. Cluster analysis was completed based on the Euclidean distance and complete linkage method.

### 2.8 Plant materials and treatments

Plump and intact mung bean seeds (cultivar VC1973A) were selected. After soaking in sterile water, the seeds were pre-germinated in darkness at 25 °C until radicle protrusion. The germinated seedlings were transplanted into a 1/2-strength MS hydroponic system and cultivated at a constant temperature of 24 °C under a 16 h light/8 h dark photoperiod; the nutrient solution was replaced regularly to ensure sufficient nutrient supply. When the first trifoliate leaf of the seedlings was fully expanded, simulated drought stress was imposed using 15% (w/v) PEG-6000 solution. Leaf samples were collected at 0, 12 h, 1 d, and 3 d post-treatment. For each sample, leaves harvested from three seedlings were mixed together, instantly frozen in liquid nitrogen, and preserved at −80 °C for later RNA extraction and gene expression analysis.

### 2.9 RNA isolation and gene expression analysis

Total RNA was isolated from all samples with a Plant Total RNA Extraction Kit (Tiangen Biotech, China). Using the PrimeScript™ RT Reagent Kit (Takara, Japan), first-strand complementary DNA (cDNA) was reverse-transcribed from the extracted total RNA. Gene-specific quantitative primers were designed for the *VrCIPK* genes, with primer sequences listed in **Supplementary Table S1**. Quantitative real-time reverse transcription PCR (qRT-PCR) was conducted using a SYBR Green-based qPCR Kit (Tiangen Biotech, China). The thermal cycling conditions were set as follows: an initial denaturation at 95 °C for 2 min, followed by 40 cycles of denaturation at 94 °C for 10 s and combined annealing-extension at 60 °C for 30 s. The *VrActin* (Vradi03g00210) gene was used as the internal reference. The relative expression levels of *VrCIPK* were determined using the 2^−ΔΔCt^ method (Livak and Schmittgen, 2001). All qRT−PCR analyses were performed with three independent biological replicates and three technical replicates per biological sample.

### 2.10 Plant transformation and generation of transgenic plants

The coding sequence (CDS) of *VrCIPK5* was amplified by PCR using cDNA as the template—cDNA was synthesized from total RNA extracted from mung bean leaves treated with 15% PEG 6000 (drought simulation). Specific primers (VrCIPK5-F/VrCIPK5-R) are listed in **Supplementary Table S1**. The CDS of *VrCIPK5* was inserted into the pCAMBIA2300 vector under the control of the CaMV 35S promoter, and the recombinant plasmid was introduced into *Agrobacterium tumefaciens* EHA105. Tobacco (*Nicotiana tabacum*) transformation was conducted using the Agrobacterium-mediated leaf disc protocol, followed by callus induction, differentiation, and rooting. Positive transformants were identified by PCR and kanamycin resistance screening (100 mg/L on MS medium). T_1_ seeds from positive plants were sown in sterilized substrate (nutrient soil: vermiculite = 2:1, v/v) after surface sterilization, and T_2_ seeds were harvested at maturity. *VrCIPK5* transcript abundance in T_2_ seedlings was verified by reverse transcription polymerase chain reaction (RT-PCR).

### 2.11 Stress resistance analysis of transgenic lines

For drought stress tolerance assays, 5-week-old seedlings with uniform growth were subjected to water withholding, while control plants were watered normally. When phenotypic differences appeared between control and transgenic plants, plants were photographed, and leaf samples were collected to determine the contents of hydrogen peroxide (H_2_O_2_), malondialdehyde (MDA), proline, and the activities of superoxide dismutase (SOD), peroxidase (POD), and catalase (CAT), as well as the expression levels of drought-responsive genes. All abiotic stress tolerance assays were performed in triplicate.

### 2.12 Determination of stomatal aperture, H_2_O_2_ accumulation, antioxidant enzyme activities, MDA and proline contents

Leaves at the same position of seedlings were used as experimental materials for physiological index measurements. Leaves were incubated in stomatal opening buffer (10 mM MES, 50 mM KCl, pH 5.8) under light to induce stomatal opening, followed by treatment with PEG-6000 solution for simulated drought stress, with sterile water as the control. The lower epidermis was stripped and mounted on slides, and stomatal morphology was observed using a BX63 microscope (Olympus, Japan). Stomatal aperture was quantified using ImageJ software (National Institutes of Health, USA). Three plants were randomly selected per line, and 20–30 stomata were measured per plant. The contents of proline, MDA, H_2_O_2_, and the activities of SOD, CAT, and POD were determined by spectrophotometry using commercial detection kits (Solarbio Science & Technology Co., Ltd., Beijing, China). Histochemical staining for H_2_O_2_ was also performed using the corresponding kit following the manufacturer’s instructions.

### 2.13 Expression analysis of stress-related genes

When phenotypic differences emerged between control and transgenic plants, leaf samples were harvested, and qRT-PCR was used to analyze the expression levels of stress-responsive genes (*NtSOD*, *NtCAT*, *NtPOX2*, *NtP5CS1*, *NtLEA5*, and *NtRD29A*). The tobacco actin gene (*NtActin*) was employed as the internal reference. All primer sequences are listed in **Supplementary Table S1**.

### 2.14 Statistical analysis

Statistical analysis was performed using GraphPad Prism 7. All experiments were conducted with at least three biological replicates. Differences among groups were evaluated by one-way analysis of variance (ANOVA) combined with Duncan’s multiple range test. A *p*-value < 0.05 was regarded as statistically significant (*), while *p* < 0.01 represented an extremely significant difference (**).

## 3 Results

### 3.1 Identification and characterization of the *VrCIPK* gene family

In this study, 23 *VrCIPK* genes were identified and named *VrCIPK1*-*VrCIPK23* based on their chromosomal locations. These genes exhibited an uneven genomic distribution, with Chromosomes 2, 4, and 10 each containing 2 genes, Chromosomes 5 and 7 each harboring 3 genes, Chromosome 6 having the most genes (4), and the remaining 7 genes (*VrCIPK17*, *-18*, *-19*, *-20*, *-21*, *-22*, *-23*) mapping to unanchored scaffolds (**Supplementary Figure S1**). The physicochemical properties of the VrCIPK proteins were analyzed. The aa lengths of the encoded proteins ranged from 325 (VrCIPK20) to 568 aa (VrCIPK10), with predicted molecular weights between 37.25 (VrCIPK20) and 64.38 kDa (VrCIPK10), and pI from 5.59 (VrCIPK3) to 9.54 (VrCIPK10). Subcellular localization prediction revealed that most VrCIPK proteins were predicted to the cytoplasm and chloroplasts. Specifically, VrCIPK4, -6, and -8 were predicted to target the plasma membrane, VrCIPK13 and -21 the nucleus, and VrCIPK14 peroxisomes (**Table 1**).

**Table 1:** Genomic information and protein characteristics of *VrCIPK* gene family.

Gene name	Gene ID	Chromosome:location	CDS (bp)	AA	Molecular weight (kDa)	PI	Subcellular localization
*VrCIPK1*	Vradi02g12930	2:23195659-23197008	1350	449	50.58	8.65	chloroplast
*VrCIPK2*	Vradi02g12950	2:23215017-23217813	1653	550	61.25	6.62	chloroplast
*VrCIPK3*	Vradi04g05680	4:12140927-12142243	1317	438	48.86	5.59	cytoplasm
*VrCIPK4*	Vradi04g05740	4:12259326-12261021	1632	543	61.22	7.86	plasma membrane
*VrCIPK5*	Vradi05g00440	5:481681-485204	1023	340	38.71	7.05	cytoplasm
*VrCIPK6*	Vradi05g03050	5:3886695-3888824	1245	414	46.86	9.37	plasma membrane
*VrCIPK7*	Vradi05g08230	5:15985408-15991406	1092	363	41.61	8.92	cytoplasm
*VrCIPK8*	Vradi06g03130	6:3332528-3339068	1290	429	48.71	6.66	plasma membrane
*VrCIPK9*	Vradi06g06520	6:8621887-8623547	1329	442	49.29	7.99	chloroplast
*VrCIPK10*	Vradi06g06540	6:8644609-8648003	1707	568	64.38	9.54	chloroplast
*VrCIPK11*	Vradi06g08160	6:14508862-14518282	1308	435	49.53	6.11	cytoplasm
*VrCIPK12*	Vradi07g20270	7:42564572-42573253	1242	413	46.94	9.28	cytoplasm
*VrCIPK13*	Vradi07g21990	7:44870006-44874208	1488	495	56.46	8.81	nucleus
*VrCIPK14*	Vradi07g24080	7:47334977-47338962	1269	422	47.59	8.26	proxisome
*VrCIPK15*	Vradi10g10020	10:17651587-17653612	1428	475	53.41	7.98	chloroplast
*VrCIPK16*	Vradi10g10030	10:17661726-17668915	1455	484	54.33	5.65	chloroplast
*VrCIPK17*	Vradi0158s00560	scaffold_158:573057-577158	1677	558	62.92	8.42	cytoplasm
*VrCIPK18*	Vradi0161s00370	scaffold_161:551558-553087	1530	509	57.50	8.00	chloroplast
*VrCIPK19*	Vradi0179s00380	scaffold_179:225666-230961	1302	433	48.98	8.79	chloroplast
*VrCIPK20*	Vradi0222s00070	scaffold_222:259848-272339	978	325	37.25	8.50	chloroplast
*VrCIPK21*	Vradi0305s00050	scaffold_305:52245-55728	1404	467	52.98	8.69	nucleus
*VrCIPK22*	Vradi0354s00050	scaffold_354:63950-67145	1149	382	43.17	9.11	cytoplasm
*VrCIPK23*	Vradi0401s00050	scaffold_401:109781-114688	1305	434	48.67	5.89	cytoplasm

### 3.2 Phylogenetic and synteny analysis of the *CIPK* gene family

**Figure 1:**
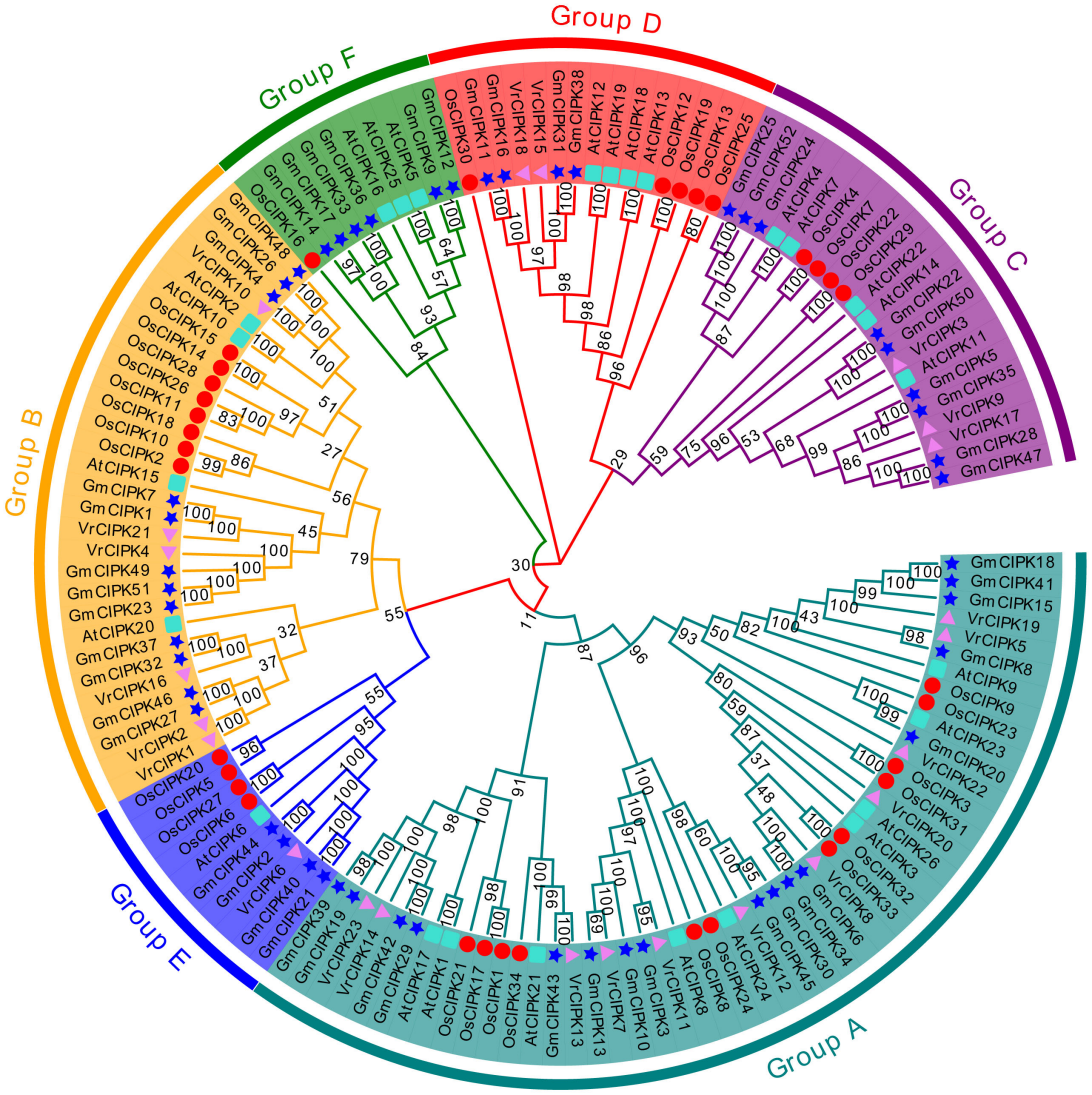
Phylogenetic analysis of CIPK proteins from mung bean, *Arabidopsis thaliana*, rice and soybean. The phylogenetic tree was constructed based on full-length amino acid sequences. All CIPK proteins were classified into six distinct groups (Groups A–F), highlighted by colored arcs. CIPK from different species are marked with unique symbols: VrCIPK (pink triangle), AtCIPK (cyan square), OsCIPK (red circle), and GmCIPK (blue star).

To elucidate the evolutionary relationships between *VrCIPK* genes and their homologs in other plant species, an unrooted phylogenetic tree was constructed based on the aa sequences of CIPK proteins from *V. radiata*, *A. thaliana*, *Oryza sativa*, and *Glycine max* (**Supplementary Table S2**). As shown in **Figure 1**, a total of 135 *CIPK* family members clustered into six groups (A–F). Among these, subgroup A contained the most *VrCIPK* members (11), and subgroup E had the fewest (1). Groups B, C, and D contained 6, 3, and 2 *VrCIPK* proteins, respectively, and no *VrCIPK* genes were assigned to subgroup F.

**Figure 2:**
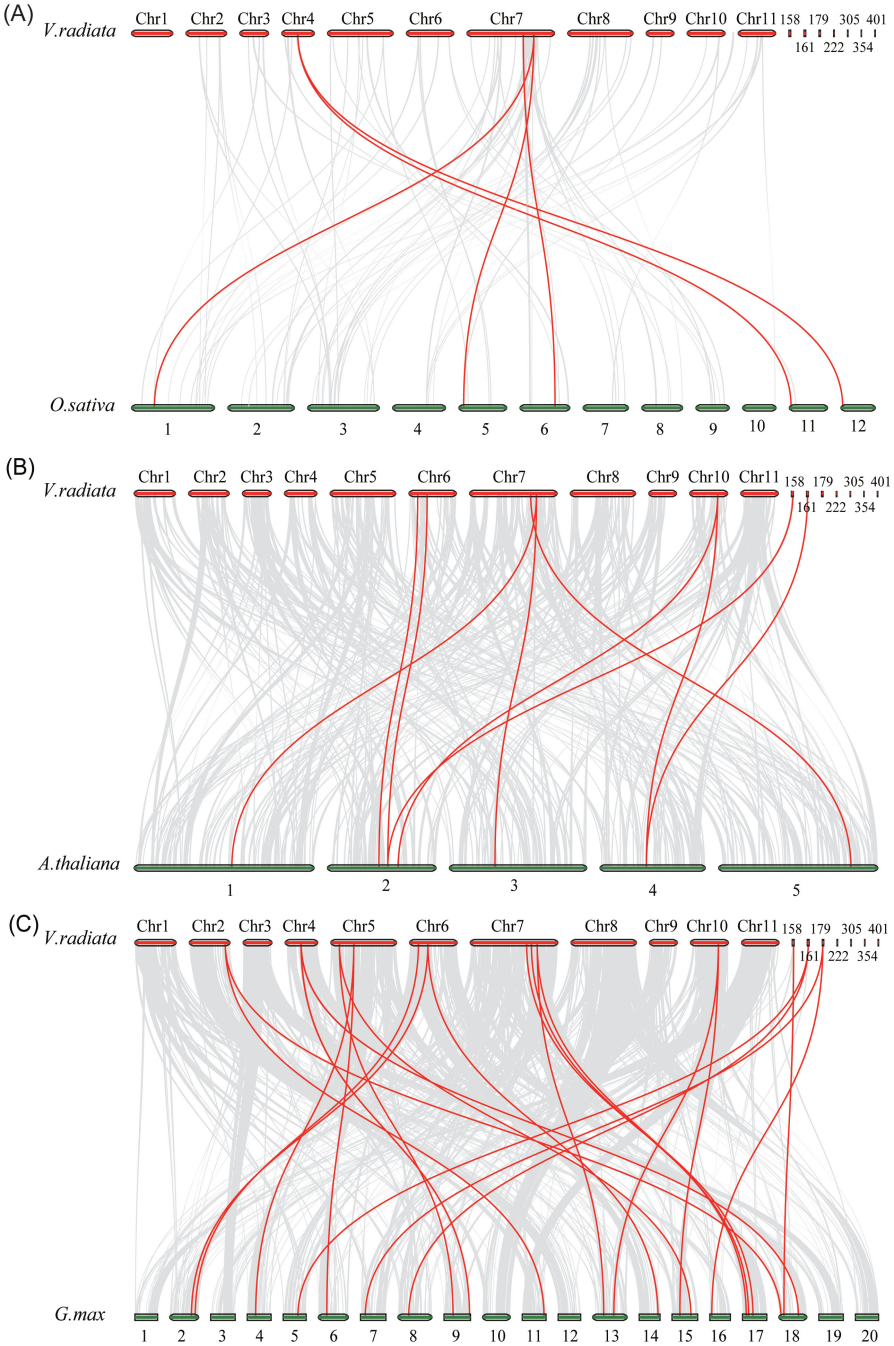
Synteny analysis of CIPK genes across different plant species. Synteny relationships of *CIPK* genes are shown between mung bean and three other species: **(A)** rice (*Oryza sativa*), **(B)** *Arabidopsis thaliana*, **(C)** soybean (*Glycine max*). Gray lines indicate whole-genome collinear blocks, and red lines denote syntenic CIPK gene pairs. Chromosomes (and unanchored scaffolds for *V. radiata*) are labeled with their respective numbers.

Synteny analysis of the *CIPK* gene family was performed in mung bean, the monocot *O. sativa*, and dicots *A. thaliana* and *G. max*, and synteny maps were generated accordingly. A total of 27 syntenic genes pairs were identified between mung bean and *G. max*, 9 between mung bean and *A. thaliana*, and 5 between mung bean and *O. sativa*. Most *VrCIPK* genes (*VrCIPK1*, *-3*, *-4*, *-6*, *-7*, *-9*, *-14*, *-15*, *-16*, *-18*, and *-19*) had syntenic relationships with two *GmCIPK* genes, and *VrCIPK8*, -*10*, *-12*, *-13*, and *-17* were syntenic with only one *GmCIPK* gene. No syntenic fragments corresponding to *VrCIPK2*, -*5*, *-11*, *-20*, *-21*, *-22*, or *-23* were detected between the mung bean and *G. max* genomes. Additionally, *VrCIPK14* exhibited syntenic relationships with *O. sativa*, *A. thaliana*, and *G. max* genomes, and *VrCIPK8*, *-9*, *-13*, *-15*, -*17*, and -*18* were syntenic with *A. thaliana* and *G. max* genomes (**Figure 2**; **Supplementary Table S3**).

### 3.3 Gene duplication analysis of *VrCIPK* genes in mung bean

**Figure 3:**
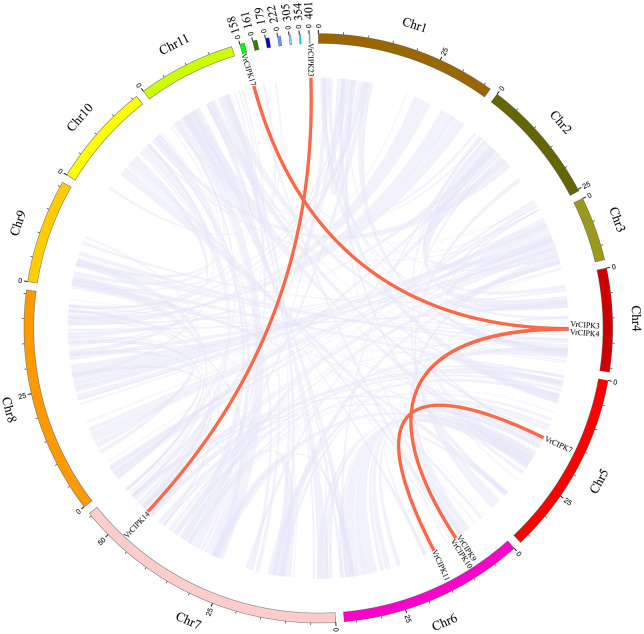
Intragenomic synteny analysis of the *CIPK* gene family in mung bean. The outer colored bars represent the 11 mung bean chromosomes (Chr1–Chr11) and scaffolds, with scale bars indicating physical positions in megabases (Mb). Light gray lines in the inner circle denote whole-genome collinear blocks identified throughout the mung bean genome, while orange lines highlight the syntenic *VrCIPK* gene pairs.

Gene duplication events were analyzed to reveal the evolutionary history of the *VrCIPK* gene family. A total of five duplication events were detected among the 23 *VrCIPK* genes, all of which were segmental duplications (no tandem duplications were identified). The five syntenic gene pairs were *VrCIPK4*/*VrCIPK10*, *VrCIPK7*/*VrCIPK11*, *VrCIPK23*/*VrCIPK14*, *VrCIPK17*/*VrCIPK3*, and *VrCIPK3*/*VrCIPK9* (**Figure 3**). Furthermore, the Ka, Ks, and Ka/Ks ratio were calculated using KaKs Calculator 2.0. Most *VrCIPK* homologous gene pairs were under strong purifying selection. Only *VrCIPK4*/*VrCIPK10* exhibited a Ka/Ks ratio > 1, indicating positive selection (**Table 2**). This suggested that *VrCIPK4* and *VrCIPK10* underwent functional changes during adaptive evolution to respond to specific selection pressures.

**Table 2:** Ka/Ks analysis for duplicated gene pairs of *CIPK* in mung bean.

Duplicated gene 1	Duplicated gene 2	Type of duplication	Ka	Ks	Ka/Ks	Purifying selection
VrCIPK4	VrCIPK10	segmental	0.614959	0.346216	1.77623	No
VrCIPK7	VrCIPK11	segmental	0.0798057	0.791059	0.100885	Yes
VrCIPK23	VrCIPK14	segmental	0.165094	0.791053	0.208702	Yes
VrCIPK17	VrCIPK3	segmental	0.486825	0.494752	0.983978	Yes
VrCIPK3	VrCIPK9	segmental	0.381383	1.67098	0.228239	Yes

### 3.4 Analysis of VrCIPK domains, motifs, and gene structures

**Figure 4:**
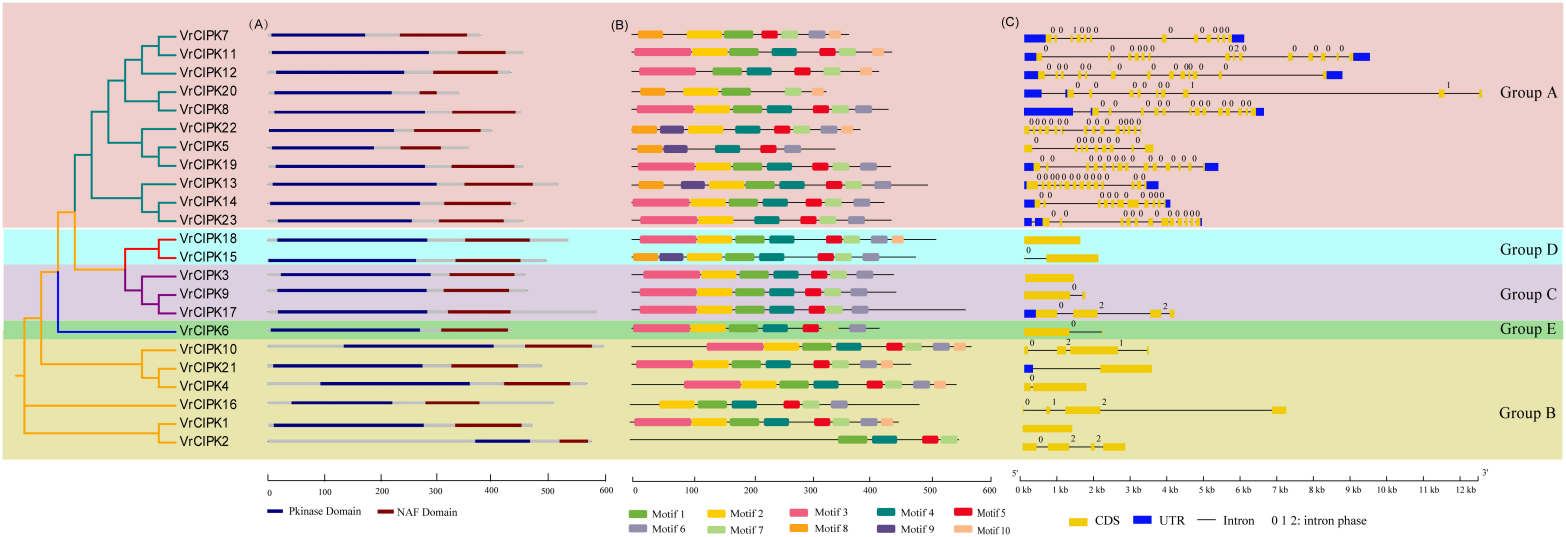
Conserved domain, motif composition, and gene structure analysis of *VrCIPK* genes. **(A)** Conserved domain architecture of VrCIPK proteins. Blue boxes represent the Pkinase domain, and red boxes represent the NAF domain, with the scale bar indicating amino acid (aa) length. **(B)** Conserved motif distribution of VrCIPK proteins. Ten conserved motifs (Motif 1–Motif 10) are represented by differently colored boxes, as detailed in the legend. **(C)** Gene structure of *VrCIPK* genes. Yellow boxes denote coding sequences (CDS), blue boxes denote untranslated region (UTR), black lines denote introns, and the numbers 0, 1, and 2 indicate the intron phase, with the scale bar showing genomic length in kilobases (kb).

Systematic analyses of the domain distribution, conserved motifs, and gene structures were performed to characterize the structural features of *VrCIPK* genes. Domain analysis revealed that all VrCIPK proteins contained an N-terminal kinase domain and a C-terminal NAF domain (**Figure 4A**; **Supplementary Table S4**). Conserved motif analysis of VrCIPK proteins using the MEME online tool identified 10 major conserved motifs. Motif 1 contained the APE residue, Motif 2 contained the DFG residue, and Motif 5 was annotated as the NAF/FISL motif (**Supplementary Figure S2**). VrCIPK in the same subgroup exhibited significant conservation in motif composition; for example, members of subgroup C shared an identical motif composition and arrangement pattern, reflecting their high evolutionary homology. Some motifs displayed a subgroup-specific distribution; for example, Motif 9 was only present in partial VrCIPK proteins of groups A and D, suggesting their potential functional divergence between groups. Additionally, motif composition differences were observed in the same subgroup, as VrCIPK2 in subgroup B lacked Motif 6, and both VrCIPK2 and VrCIPK16 lacked Motif 10, indicating the functional specialization of VrCIPK in the same subgroup (**Figure 4B**).

Gene structure analysis showed that the 11 *VrCIPK* genes in subgroup A contained 8–13 introns, and those in subgroups B–F contained no more than three introns. Notably, *VrCIPK1*, *VrCIPK3*, and *VrCIPK18* lacked introns. In subgroup A, all genes except *VrCIPK11* and *VrCIPK20* had an intron phase of zero, indicating high conservation in intron position and phase patterns. Combined with phylogenetic analysis, *VrCIPK* genes with close evolutionary relationships exhibited a highly conserved number of introns and phase characteristics (**Figure 4C**).

### 3.5 GO functional enrichment analysis of *VrCIPK* genes

To systematically dissect the biological functions of VrCIPK family proteins, GO enrichment analysis was conducted on all 23 *VrCIPK* genes, and a chord diagram was generated to visualize gene–GO term correlations (**Figure S5**; **Supplementary Table S5**). All genes were annotated into three standard GO categories: Biological Process (BP), Molecular Function (MF), and Cellular Component (CC). In BP, protein phosphorylation and signal transduction dominated the enriched terms, representing core plant calcium signaling cascades. The top MF terms were protein kinase activity and ATP binding, which jointly support the catalytic function of CIPK. As serine/threonine kinases, all VrCIPK proteins depend on ATP binding to activate kinase activity and propagate calcium signals, highlighting conserved catalytic capacity across the family. For CC, VrCIPK proteins predominantly localize to the cytoplasm, nucleus, membrane and vacuole. Most members exhibit dual cytoplasmic-nuclear distribution, facilitating calcium signal transmission from membrane sensors to nuclear transcription factors. Overall, GO enrichment results confirm that VrCIPK mediate calcium signal transduction via conserved kinase functions.

**Figure 5:**
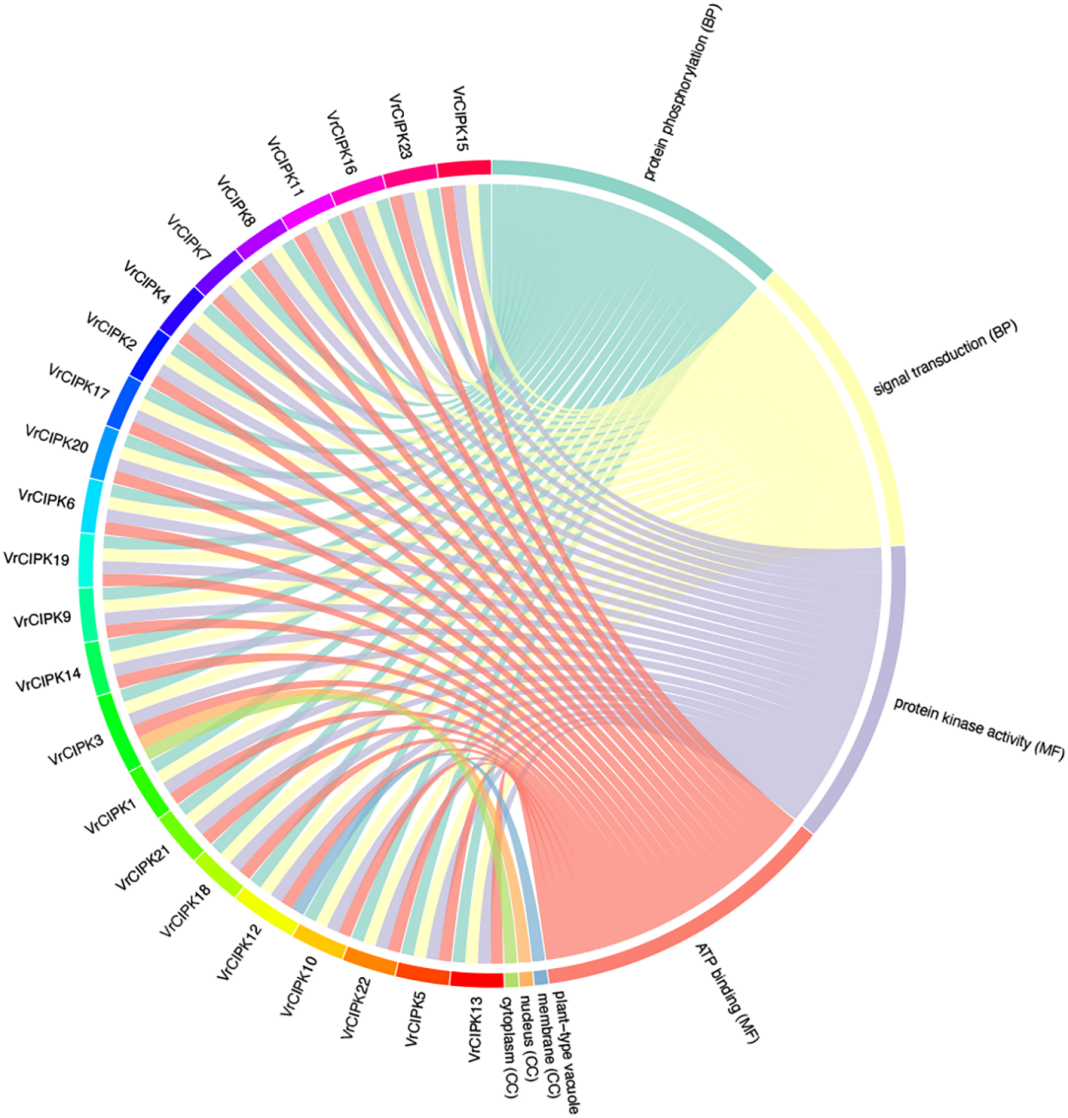
GO enrichment chord diagram of all *VrCIPK* genes. Chords represent the correlation between individual *VrCIPK* genes and core enriched GO terms, which are divided into three categories: Biological Process (BP), Molecular Function (MF), and Cellular Component (CC).

### 3.6 Analysis of cis-acting elements in the promoters of *VrCIPK* genes

**Figure 6:**
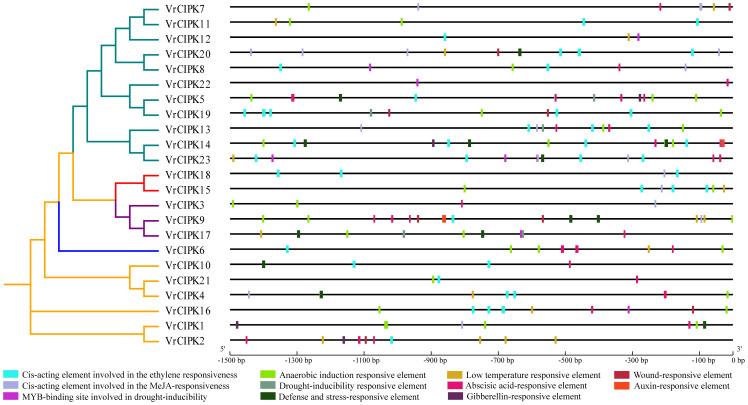
Cis-acting element analysis in the promoter regions of *VrCIPK* genes in mung bean. The scale bar at the bottom indicates the length of the promoter sequence. Different colored boxes represent cis-acting elements with distinct functions, as detailed in the legend.

Cis-acting elements were predicted in the 1500-bp upstream region of ATG for each *VrCIPK* gene. The promoter regions of the *VrCIPK* genes were enriched in hormone-responsive and abiotic stress-responsive elements. Among the hormone-responsive elements, ABA-responsive elements, methyl jasmonate-responsive elements, and ethylene-responsive elements were the most widely distributed, and auxin-responsive elements and gibberellin-responsive elements were only present in a few *VrCIPK* genes. For abiotic stress-responsive elements, anaerobic induction elements were detected in almost all *VrCIPK* genes, and low-temperature, drought, wounding, and defense-related stress-responsive showed a gene-specific distribution. MYB binding sites associated with drought induction were identified in multiple *VrCIPK* genes (**Figure 6**; **Supplementary Table S6**). Divergence was observed in the cis-acting element distribution within the same subgroup, indicating the functional gene specialization within the subgroup.

### 3.7 Expression analysis of *VrCIPK* genes under drought stress

**Figure 7:**
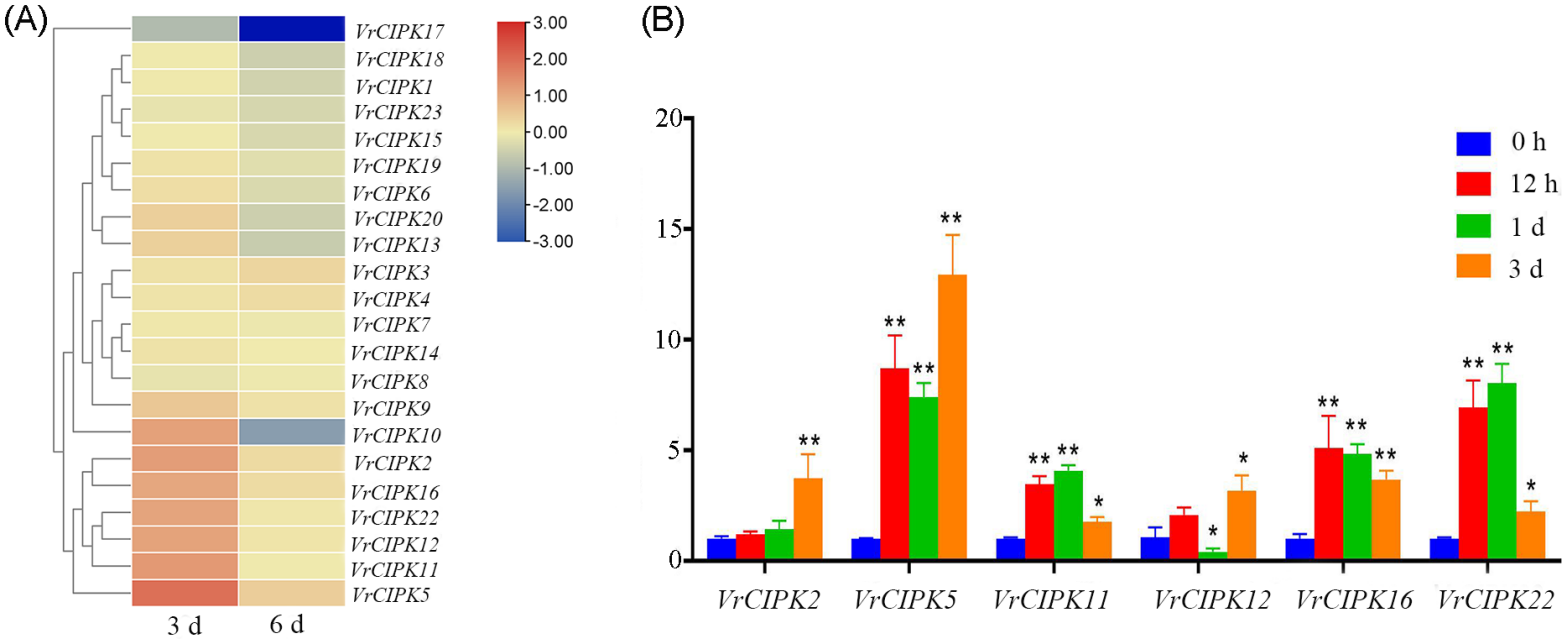
Drought stress-induced expression analysis of *VrCIPK* genes and qRT-PCR validation. **(A)** Heatmap of *VrCIPK* gene expression based on transcriptome data under 3 d and 6 d drought stress. The color scale represents log_2_-normalized expression values (blue: downregulated, red: upregulated). **(B)** qRT-PCR validation of six differentially expressed *VrCIPK* genes at 0 h (control), 12 h, 1 d, and 3 d after drought treatment. Bars represent relative expression levels, with error bars indicating SE of three biological replicates. Asterisks indicate significant differences vs. the 0 h control (*P < 0.05, **P < 0.01).

The expression profiles of *VrCIPK* genes in mung bean leaves under drought stress were analyzed using RNA sequencing (RNA-seq) data from the NCBI SRA database to clarify the function of the *VrCIPK* gene family in drought stress responses. Differential expression analysis revealed that some genes, including *VrCIPK5* and *VrCIPK11* were significantly upregulated under drought stress, while others, including *VrCIPK7* and *VrCIPK14*, showed no significant expression changes. *VrCIPK17* was significantly downregulated (**Figure 7A**). The TPM of *VrCIPK21* was zero in all biological replicates of the control and drought-treated groups; thus, this gene was not displayed in the heatmap. To verify the reliability of the RNA-seq data, differentially expressed *VrCIPK* genes with Log_2_FC > 1 were selected for validation with quantitative real-time PCR (qRT-PCR). The results showed that *VrCIPK2*, *VrCIPK5*, *VrCIPK11*, *VrCIPK12*, *VrCIPK16*, and *VrCIPK22* were upregulated under drought stress, consistent with the RNA-seq results. Among these validated genes, *VrCIPK5* exhibited the highest relative expression level (**Figure 7B**).

### 3.8 Phenotypic and physiological responses of *VrCIPK5*-overexpressing tobacco

**Figure 8:**
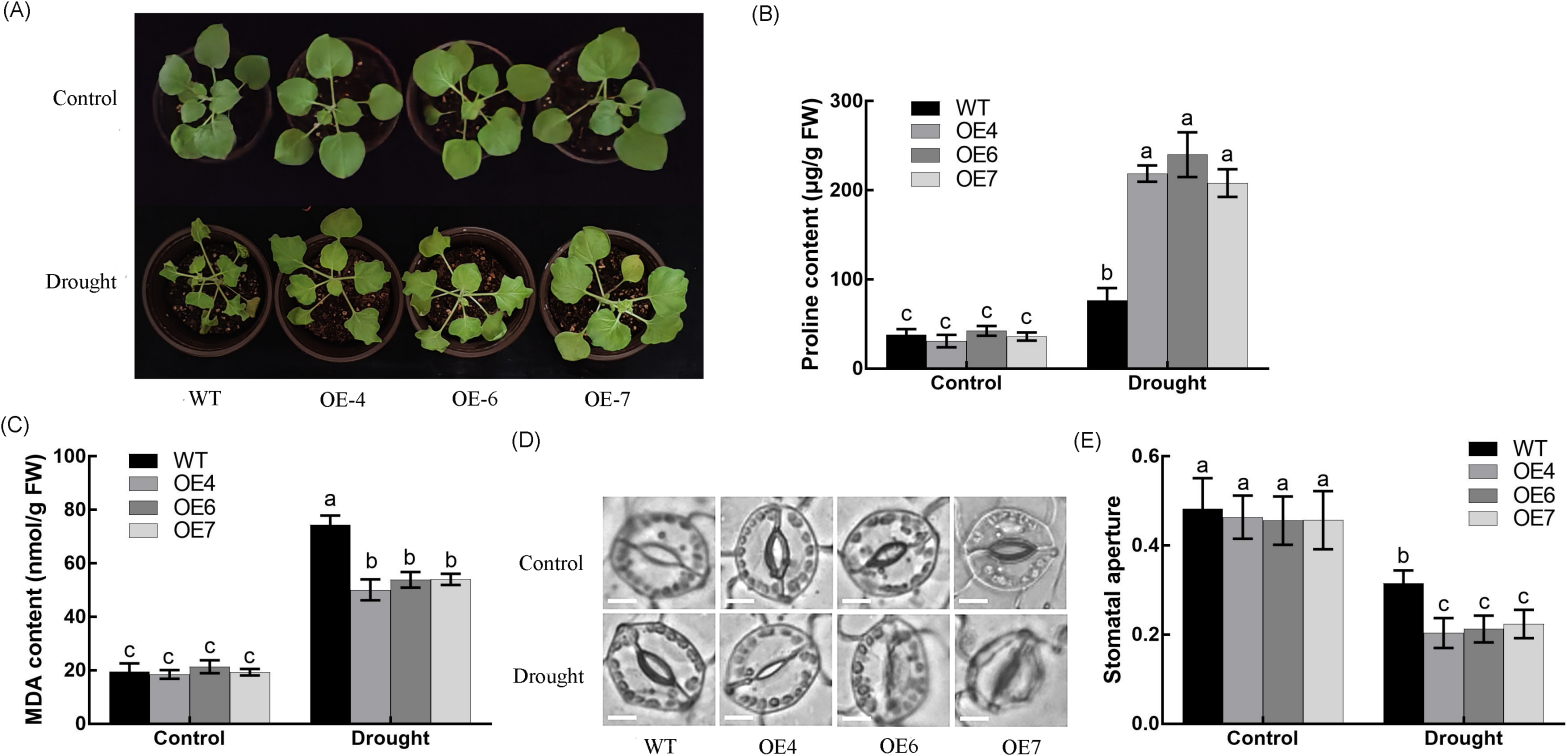
Drought tolerance analysis of *VrCIPK5* overexpression transgenic plants. **(A)** Phenotypes of WT and three independent *VrCIPK5* overexpression (OE) lines under control and drought stress conditions. **(B)** Proline content, **(C)** MDA content in leaves of WT and OE lines under control and drought stress. **(D)** Representative stomatal images of WT and OE lines under normal and drought conditions. Scale bars = 10 μm. **(E)** Statistical analysis of stomatal aperture (width/length ratio) in WT and OE lines under control and drought stress. Data are presented as means ± SD from three biological replicates. Different lowercase letters indicate significant differences among groups (*P* < 0.05, one-way ANOVA followed by Duncan’s test).

Previous studies have shown that the *CIPK* gene family participates in plant calcium signaling and is critical for drought stress responses (Chaves-Sanjuan et al., 2014). In this study, *VrCIPK5* was significantly induced by drought. *VrCIPK5*-OE transgenic tobacco lines were generated in this study. PCR and RT-PCR verified gene integration and expression (**Supplementary Figure S3**). Three high-expression lines (OE4, OE6, and OE7) were selected for functional analysis. Five-week-old transgenic and wild-type (WT) tobacco plants were subjected to natural drought stress. No significant differences in growth phenotypes were observed between WT and OE lines before drought stress. After 15 days of drought treatment, WT showed wilting and leaf curling, and OE lines only exhibited slight wilting symptoms (**Figure 8A**). To elucidate the physiological mechanisms underlying these phenotypic differences, the proline content, MDA content, and stomatal aperture were measured after drought stress. Drought stress significantly increased the proline content in all tested lines, with OE lines exhibiting higher levels than WT (**Figure 8B**). The MDA content in WT was significantly higher than that in OE lines (**Figure 8C**). Stomatal transpiration is the main pathway of plant water loss. Under light conditions, leaf stomata were fully open in all lines in the buffer solution. After dehydration in air for 2 h, the stomatal aperture of OE lines was significantly smaller than that of WT (**Figures 8D, E**).

### 3.9 Reactive oxygen species scavenging capacity of *VrCIPK5*-OE tobacco

**Figure 9:**
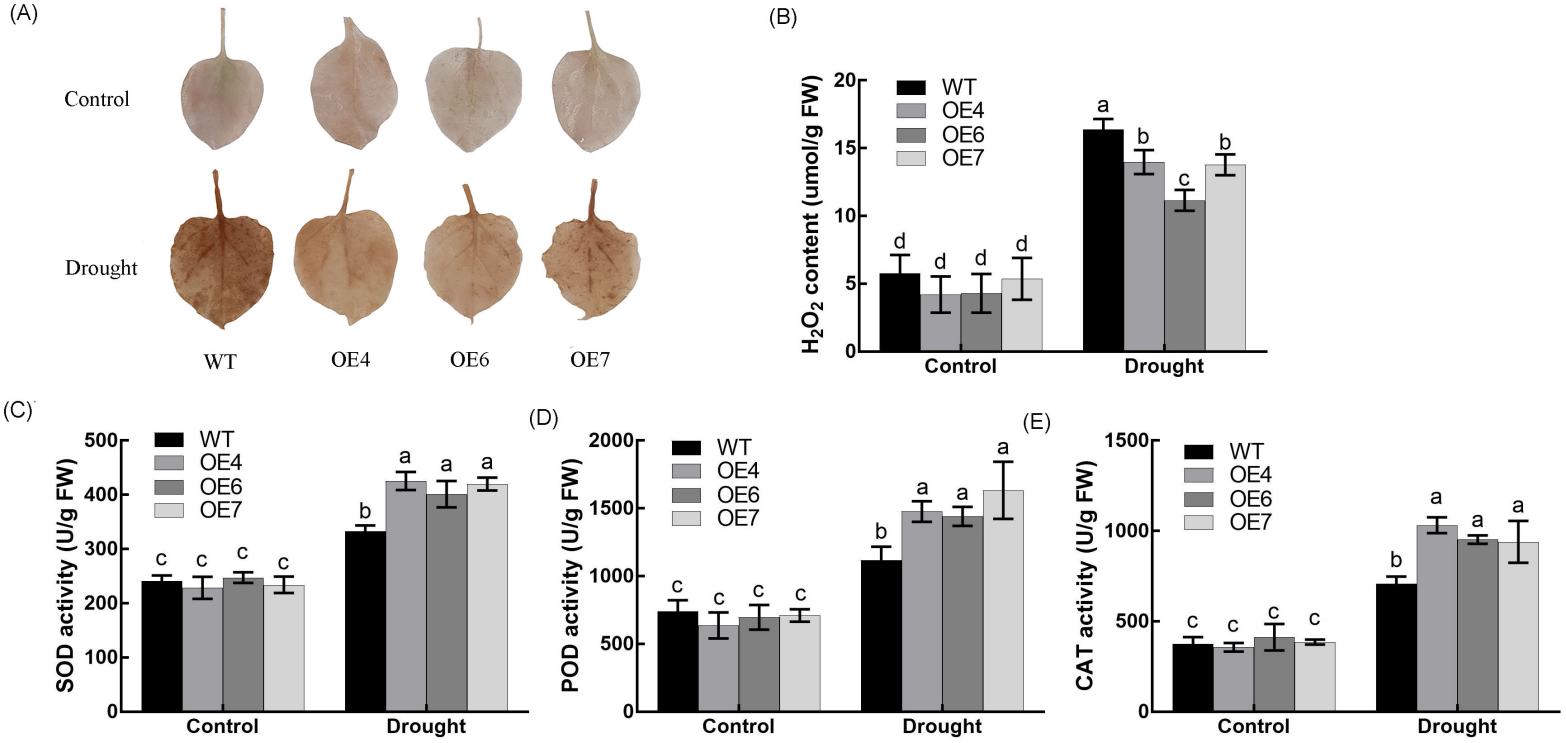
Drought-induced changes in reactive oxygen species (ROS) accumulation and antioxidant enzyme activities in wild-type (WT) and *VrCIPK5* overexpression (OE) lines. **(A)** Phenotypic comparison of H_2_O_2_-induced leaf color changes in WT and three OE lines (OE4, OE6, OE7) under normal growth (Control) and drought stress conditions. **(B)** H_2_O_2_ content, **(C)** SOD activity, **(D)** POD activity, and **(E)** CAT activity in leaves of WT and OE lines under control and drought stress. Data are presented as means ± SD from three biological replicates. Different lowercase letters indicate significant differences among groups (*P* < 0.05, one-way ANOVA followed by Duncan’s test).

In this study, ROS accumulation in tobacco leaves was detected after drought treatment using 3,3’-diaminobenzidine staining. The staining intensity of *VrCIPK5*-OE lines was significantly lighter than that of WT, indicating that the accumulation of H_2_O_2_ and superoxide anions in the leaves of OE lines was significantly lower than that in WT (**Figure 9A**). The quantitative determination of the leaf H_2_O_2_ content confirmed the staining results (**Figure 9B**). The plant ROS scavenging enzyme system eliminates excess ROS to alleviate oxidative damage. In this study, SOD, POD, and CAT were selected as representative ROS scavenging enzymes, and their activities were measured after drought treatment. The SOD, POD, and CAT activities were significantly higher in OE lines than in WT under drought stress (**Figures 9C–E**).

### 3.10 Regulation of stress-responsive gene expression in *VrCIPK5*-OE tobacco

**Figure 10:**
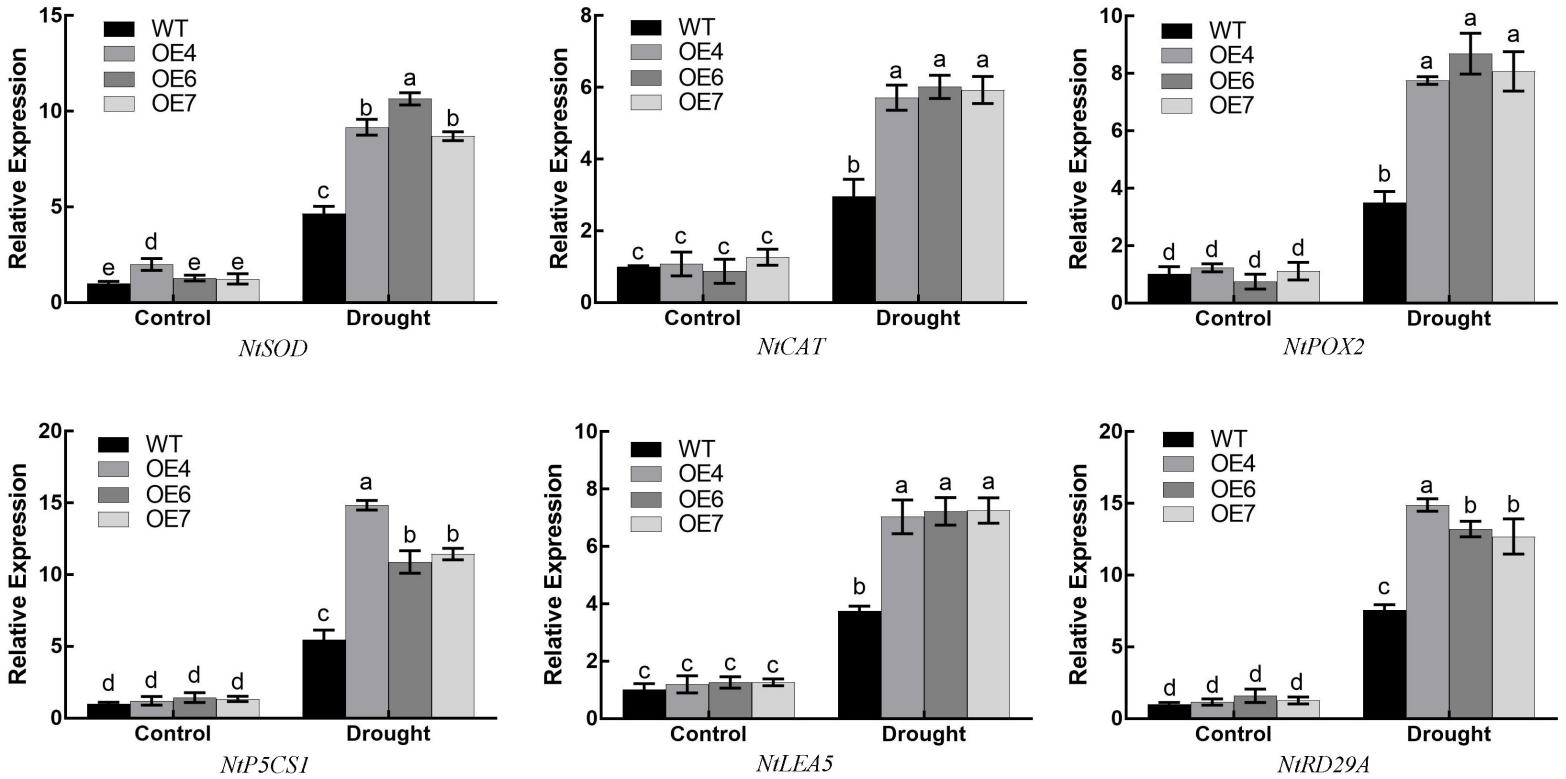
Expression analysis of antioxidant and stress-related genes in transgenic and wild-type plants under drought stress. Gene expression was normalized against *Actin* as the internal reference gene. Data are presented as means ± SD from three biological replicates. Different lowercase letters indicate significant differences among groups (*P* < 0.05, one-way ANOVA followed by Duncan’s test).

To reveal the molecular mechanism by which *VrCIPK5* regulates drought tolerance, the transcriptional levels of representative stress-responsive genes were detected in tobacco under drought stress. Among these genes, *NtSOD*, *NtCAT*, and *NtPOX2* encode antioxidant enzymes, which alleviates oxidative damage by scavenging intracellular ROS (Zhao et al., 2020). *NtP5CS1* encodes a rate-limiting enzyme for proline synthesis, promoting proline accumulation and enhancing the osmotic adjustment capacity (Hong et al., 2000). *NtLEA5* belongs to the late embryogenesis abundant (LEA) protein family, which maintains cell membrane and biomacromolecule stability during cell dehydration (Amara et al., 2012). *NtRD29A* is a typical drought-responsive marker gene that rapidly responds to drought signals and initiates downstream stress defense pathways (Yamaguchi-Shinozaki and Shinozaki, 1993). There were no significant differences in the expression of the detected genes between WT and *VrCIPK5*-transgenic tobacco under normal conditions. After drought treatment, all six genes were significantly upregulated in all lines, and their expression levels were significantly higher in OE lines than in WT (**Figure 10**).

## 4 Discussion

As a core regulatory component of calcium signaling pathways, the *CIPK* gene family plays pivotal roles in plant growth, development, and abiotic stress responses (Pandey, 2008). Although the *CIPK* gene family has been systematically identified in various plant species, the systematic identification and stress-responsive functional characterization of the *CIPK* gene family remain unreported in mung bean. In this study, 23 *VrCIPK* genes were identified in the mung bean genome using bioinformatics approaches. These genes exhibited an uneven chromosomal distribution, indicating that they underwent chromosomal segment rearrangement and integration events during evolution (Adams and Wendel, 2005; Vicient and Casacuberta, 2017). Significant variations were observed in the aa length, molecular weight, and pI of VrCIPK proteins. Subcellular localization prediction revealed that these proteins were predicted to be widely distributed across subcellular compartments, including the cytoplasm, chloroplasts, plasma membrane, nucleus, and peroxisomes. This variation in physicochemical properties and subcellular localization suggests that VrCIPK proteins participate in physiological processes in different subcellular regions in mung bean. The number of *VrCIPK* genes identified in mung bean (23) is fewer than that reported in *A. thaliana* (26) (Kolukisaoglu et al., 2004) and rice (34) (Kanwar et al., 2014). The mung bean genome spans approximately 494–579 Mb and encodes an estimated 22,368 genes (Kang et al., 2014), whereas the genome sizes of *A. thaliana* and rice are 125 and 403 Mb, respectively (Yu et al., 2002; Initiative, 2000). Despite mung bean having a larger genome, the *Vr*CIPK gene family size did not expand proportionally, indicating that the *CIPK* gene family size is not directly correlated with the genome size but may instead be associated with divergent gene duplication events during species evolution (Cao et al., 2017).

Phylogenetic tree analysis revealed high evolutionary conservation between *VrCIPK* family members and *CIPK* homologs from other plant species, with all members clustered into six groups (Groups A–F). This suggests that *CIPK* family members underwent similar evolutionary patterns. Additionally, the number of members in each subgroup varies among plant species, with *A. thaliana* having 4, 4, 3, 8, 0, and 2 *CIPK* genes, rice having 9, 8, 7, 11, 6, and 0, soybean having 7, 8, 5, 28, 7, and 4, and mung bean having 6, 3, 3, 2, 9, and 0 in Groups A-F, respectively. These results indicate that *CIPK* family members share a common ancestor and diverged during speciation. No *VrCIPK* genes were assigned to Group F, suggesting that *VrCIPK* genes in this subgroup might not have diverged or were lost in mung bean during evolution. This may be associated with the adaptive evolution of mung bean to specific environments, as a similar observation has been reported in the *CIPK* gene family of quinoa (Zhu et al., 2022).

As a key approach for genome evolution analysis, synteny analysis effectively reveals genetic relationships and functional correlations between genes across species. In this study, 27 syntenic gene pairs were identified between mung bean and the leguminous plant soybean, a number significantly higher than that between mung bean and *A. thaliana* (9 pairs) or rice (5 pairs). This indicates a closer genetic relationship between the *CIPK* genes of mung bean and soybean, consistent with the evolutionary classification of leguminous plants (Kang et al., 2014). Further analysis showed that *VrCIPK14* formed syntenic relationships with *CIPK* genes from rice, *A. thaliana*, and soybean and that six genes (*VrCIPK8*, -9, -13, -15, -17, and -18) exhibited synteny with *CIPK* genes from *A. thaliana* and soybean. These findings suggest that these genes are core conserved members of the *CIPK* family, retaining critical biological functions during long-term plant evolution (Ma et al., 2020; Kaya et al., 2024). Although most *VrCIPK* genes formed syntenic relationships with *CIPK* genes from other species, no obvious syntenic fragments were detected for seven genes (*VrCIPK2*, *-5*, -*11*, *-20*, -*21*, -*22*, and -*23*). We speculate that these are novel genes generated during the species-specific evolution of mung bean or that they have undergone significant sequence divergence over time (Yang et al., 2022). During plant evolution, gene duplication is a major mechanism for gene amplification, with segmental and tandem duplication playing key roles in the expansion of gene family members (Moore and Purugganan, 2003). The analysis of gene duplication events in *VrCIPK* genes from mung bean identified five segmentally duplicated *VrCIPK* gene pairs with no tandem duplications detected. Thus, segmental duplication is the primary mode of expansion for the mung bean *VrCIPK* gene family, consistent with the evolutionary expansion mechanisms of the *CIPK* gene family in potato and soybean (Zhu et al., 2016; Ma et al., 2021). Previous studies have demonstrated that tandem duplication is prevalent in gene families with a large number of members and high evolutionary rates, whereas segmental duplication is typical of slowly evolving gene families (Cannon et al., 2004). Accordingly, the mung bean *VrCIPK* gene family shows characteristics of slow evolution. With the exception of *VrCIPK4*/*VrCIPK10*, the *K*a/*K*s ratios of all duplicated *VrCIPK* gene pairs were less than one, indicating that most segmentally duplicated *VrCIPK* genes underwent purifying selection. Since purifying selection constrains gene divergence, this indicates that duplicated *VrCIPK* genes retain similar functions following duplication (Liu et al., 2014b).

This study systematically characterized the domain distribution, conserved motif composition, and gene structure of the *VrCIPK* gene family. VrCIPK proteins exhibited typical structural features of the plant *CIPK* gene family: kinase domain localized at the N-terminal region, and NAF domain concentrated in the C-terminal region. In the conserved N-terminal kinase domain, Motif 1 contains the APE conserved residue and Motif 2 carries the DFG conserved residue. The C-terminal region contains the characteristic NAF/FISL motif. These structural features demonstrate that the core functional structures of CIPK proteins have been highly conserved during long-term plant evolution. This conservation provides the structural basis for maintaining kinase catalytic activity and ensures the interaction specificity of CIPK proteins with other regulatory factors (Batistic and Kudla, 2004; Chaves-Sanjuan et al., 2014). Combined with the conserved structural features identified above, GO annotation of mung bean VrCIPKs provides solid molecular evidence for calcium-mediated signaling in mung bean. Terms related to signal transduction and protein phosphorylation were highly enriched in Biological Process (BP), while protein kinase activity and ATP binding dominated Molecular Function (MF), consistent with the conserved kinase domains detected in sequence analysis.

The intron–exon gene structure serves as a typical signature of gene family evolution, and variations in its distribution pattern and number can reflect the evolutionary history of the gene family (Liu et al., 2014a). The 23 *VrCIPK* genes can be divided into two categories based on the number of introns: intron-rich (containing 8–13 introns) and intron-poor (intron-less or containing ≤ 3 introns). Intron-rich genes were concentrated in Group A, and intron-poor and intron-less genes clustered in Groups B–E. This distribution pattern suggests that members in the intron-rich subfamily have closer genetic relationships, and members in the intron-poor and intron-less subfamilies exhibit similar phylogenetic characteristics. During gene segmental duplication, the rate of intron loss is faster than that of intron gain (Zhu et al., 2022). Thus, intron-rich *VrCIPK* genes may have a higher sequence similarity to the ancestral genes of the family, experiencing relatively weaker selection pressure and stronger structural conservation during evolution. Notably, *VrCIPK20* possesses a genomic length exceeding 10 kb, which is distinctly longer than other *VrCIPK* genes. This length expansion may predominantly arise from the intron expansion putatively mediated by transposable element insertion (Lee and Kim, 2014). Moreover, gene duplication events can boost structural divergence by adding extra non-coding sequences to introns (Xu et al., 2012).

Cis-acting elements in the promoter region are critical for gene transcriptional regulation, and their types and distribution determine gene expression patterns. In this study, many hormone-responsive and abiotic stress-responsive elements were identified in *VrCIPK* gene promoter regions. These elements may endow *VrCIPK* genes with the potential to respond to abiotic stressors and regulate endogenous hormones, providing a molecular basis for functional prediction. The *CIPK* gene family has been confirmed to have critical regulatory functions in plant drought stress responses (Yang et al., 2022; Chaves-Sanjuan et al., 2014). In this study, the expression profiles of the mung bean *VrCIPK* gene family were analyzed under 3 and 6 days of drought stress based on transcriptome data. Some genes, including *VrCIPK5* and *VrCIPK11*, were significantly upregulated, and others, including *VrCIPK7* and *VrCIPK14*, showed no significant expression changes. *VrCIPK17* was significantly downregulated. This suggests the functional divergence of the *VrCIPK* gene family in the drought stress responses of mung bean, consistent with the drought-responsive characteristics of *CIPK* genes in other crops, such as potato and wheat (Ma et al., 2021; Pouya et al., 2025). qRT-PCR confirmed that *VrCIPK2*, *VrCIPK5*, *VrCIPK11*, *VrCIPK12*, *VrCIPK16*, and *VrCIPK22* were significantly upregulated under drought stress, consistent with the transcriptome data. Among these validated genes, *VrCIPK5* exhibited the highest relative expression. Based on the prediction of cis-acting elements in the promoter region, the upstream regulatory region of *VrCIPK5* contained ABA-responsive and drought stress-responsive elements. We speculate that the high expression of *VrCIPK5* under drought induction may be coordinately regulated by these elements. Thus, *VrCIPK5* may play a critical role in the drought resistance physiology of mung bean. In addition, subcellular localization prediction indicated that VrCIPK5 is localized in the cytoplasm, consistent with the typical cytoplasmic distribution of many stress-related CIPK proteins. Such cytoplasmic localization enables VrCIPK5 to participate in cytosolic Ca²^+^ signal transduction, regulate cytoplasmic antioxidant enzymes, and mediate downstream drought stress responses at the cellular level (Batistic and Kudla, 2004; Ma et al., 2021; Kaya et al., 2024).

To elucidate the regulatory role of *VrCIPK5* in drought resistance, *VrCIPK5*-OE transgenic tobacco lines were generated for phenotypic analysis of drought tolerance. After 15 days of natural drought stress, phenotypic analysis showed that WT exhibited typical stress damage phenotypes, such as severe wilting and leaf curling, while *VrCIPK5*-OE lines showed slight wilting without obvious leaf curling, confirming that *VrCIPK5* overexpression significantly enhanced the drought tolerance of tobacco plants. The improved drought resistance phenotype in OE lines was closely associated with enhanced stomatal closure observed in this study. Studies have shown that as a core component of the CBL–CIPK signaling module, CIPK kinases are typically activated by ABA−triggered Ca²^+^ signals through interactions with specific CBL calcium sensors. The CBL–CIPK complex may then phosphorylate guard cell ion channels, which could promote stomatal closure to reduce water loss (Cheong et al., 2003; Luan, 2009; Ma et al., 2021; Kaya et al., 2024). In line with this regulatory mechanism, the significantly smaller stomatal aperture in *VrCIPK5*−overexpressing tobacco plants under drought suggests that *VrCIPK5* may be involved in ABA−mediated stomatal closure. Our results imply that *VrCIPK5* might act as a key node in the CBL–CIPK dependent ABA pathway to regulate stomatal movement, thus reducing water loss and ultimately enhancing plant drought resistance.

Beyond regulating stomatal movement, *VrCIPK5* also enhanced drought tolerance by improving cellular osmotic adjustment capacity. Under water deficit stress, plants actively accumulate compatible osmotic adjustment substances, such as proline, to reduce the cell osmotic potential, maintain cellular water homeostasis, and enhance adaptability to adverse environments (Verbruggen and Hermans, 2008). As a key osmotic adjustment substance, proline accumulation is positively correlated with plant drought resistance, and improves plant stress tolerance through multiple pathways, such as stabilizing biomacromolecular structures and alleviating oxidative damage (Szabados and Savouré, 2010). In this study, the leaf proline content of OE lines was significantly higher than that of WT after drought treatment, indicating that overexpression of *VrCIPK5* significantly enhanced the osmotic adjustment capacity of transgenic tobacco. Meanwhile, *VrCIPK5* overexpression effectively alleviated drought-induced oxidative damage and enhanced cellular ROS scavenging capacity. Stress can induce high ROS production in plant cells, leading to severe oxidative damage, disrupting membrane system integrity, and reducing enzyme activity (Miller et al., 2010). The accumulation of MDA, a characteristic end product of membrane lipid peroxidation, directly reflects the degree of oxidative damage to biological membranes (Mottley et al., 1991). ROS scavenging in plants depends on the activity of antioxidant enzymes, such as SOD, POD, and CAT (Mittler et al., 2004). After drought treatment, the H_2_O_2_ and MDA contents in OE lines were lower than those in WT, but the SOD, POD, and CAT activities were higher than those in WT. Consistent with the improved physiological performance, *VrCIPK5* positively regulated the transcription of multiple drought-responsive downstream genes. qRT-PCR showed that the transcriptional expression levels of *NtSOD*, *NtCAT*, *NtPOX2*, *NtP5CS1*, *NtLEA5*, and *NtRD29A* genes were significantly higher in *VrCIPK5*-OE lines than in WT after drought stress treatment. Under normal growth conditions, there were no significant differences in the expression levels of these genes between the OE lines and WT. Taken together, our results demonstrate that *VrCIPK5* enhances tobacco drought tolerance through multiple synergistic mechanisms. *VrCIPK5* reduces transpirational water loss by promoting ABA-mediated stomatal closure, strengthens osmotic adjustment by increasing proline accumulation, alleviates oxidative membrane damage by activating antioxidant systems, and up-regulates the expression of core drought-defense genes. Through these coordinated pathways, *VrCIPK5* comprehensively improves the stress resistance and adaptive capacity of tobacco under drought conditions.

Heterologous overexpression in tobacco helped validate the positive regulatory role of *VrCIPK5* under drought conditions. However, as a Solanaceae plant distantly related to mung bean, tobacco lacks the mung bean-specific CBL–CIPK regulatory network, meaning results from this heterologous system may not fully recapitulate the endogenous function of *VrCIPK5*. Even so, the mechanistic clues acquired from tobacco heterologous assays still provide valuable preliminary evidence for understanding the drought response of *VrCIPK5*. Additionally, this work only examined drought stress, the primary yield-limiting stress for mung bean in arid zones. Since *CIPK* broadly mediate salinity, heavy metal and other abiotic stress responses, future multi-stress assays will uncover the full regulatory roles of *VrCIPK* members.

## 5 Conclusion

This study conducted the first genome-wide analysis of the mung bean *CIPK* family, identifying 23 unevenly distributed *VrCIPK* genes. These genes were divided into five conserved groups, with segmental duplication driving gene expansion and most duplicated gene pairs under purifying selection. Multiple bioinformatic analyses confirmed conserved calcium signal transduction functions and abundant hormone- and stress-related cis-acting elements in *VrCIPK* promoters. Transcriptome and qRT-PCR confirmed that *VrCIPK5* is markedly induced by drought stress. Heterologous overexpression in tobacco validated that *VrCIPK5* improves drought resistance via proline accumulation, elevated antioxidant enzyme activity, accelerated stomatal closure, activated downstream stress genes, and reduced ROS and membrane lipid peroxidation. Our results fill the research blank of mung bean *CIPK* family and offer genetic materials for drought-tolerant mung bean breeding.

## Data Availability

Publicly available datasets were analyzed in this study. This data can be found here: Genome sequences and annotation files used for identifying and analyzing the *CIPK* gene family were derived from the Ensembl Plants Database (http://plants.ensembl.org/index.html). The RNA-seq datasets analyzed in this study are deposited in NCBI SRA under BioProject accession PRJNA764584 (https://www.ncbi.nlm.nih.gov/bioproject/PRJNA764584). All relevant data generated or analyzed in this study are included in this article and its supplementary information files.
