# Supplementary material for: Genome-wide characterization of the CIPK gene family in mung bean and functional validation of VrCIPK5 in drought stress response
Source: Front Plant Sci. 2026 Jul 20;17:1866081. doi: 10.3389/fpls.2026.1866081 (PMC13430420; doi:10.3389/fpls.2026.1866081)
Supplement: Supplementary file 2 [file SupplementaryFile1.docx]

**Supplementary Figure captions**

**Figure S1.** The distribution of *VrCIPK* genes on each chromosome (chr) and scaffolds based on physical position.

**Figure S2.** Sequence logos of the 10 conserved motifs identified in VrCIPK proteins. The height of each amino acid residue in motifs reflects its conservation at that position.

**Figure S3.** Validation of *VrCIPK5* overexpression in transgenic lines. **(A)** Genomic PCR confirmation of the *VrCIPK5* transgene in OE lines (OE4, OE6, OE7), with WT as negative control and M as DNA marker. **(B)** RT-PCR analysis of *VrCIPK5* expression, with *Actin* as the internal reference, demonstrating successful overexpression in transgenic lines.
