# Supplementary figures and images for: Genome-wide characterization of the CIPK gene family in mung bean and functional validation of VrCIPK5 in drought stress response
Source: Front Plant Sci. 2026 Jul 20;17:1866081. doi: 10.3389/fpls.2026.1866081 (PMC13430420; doi:10.3389/fpls.2026.1866081)

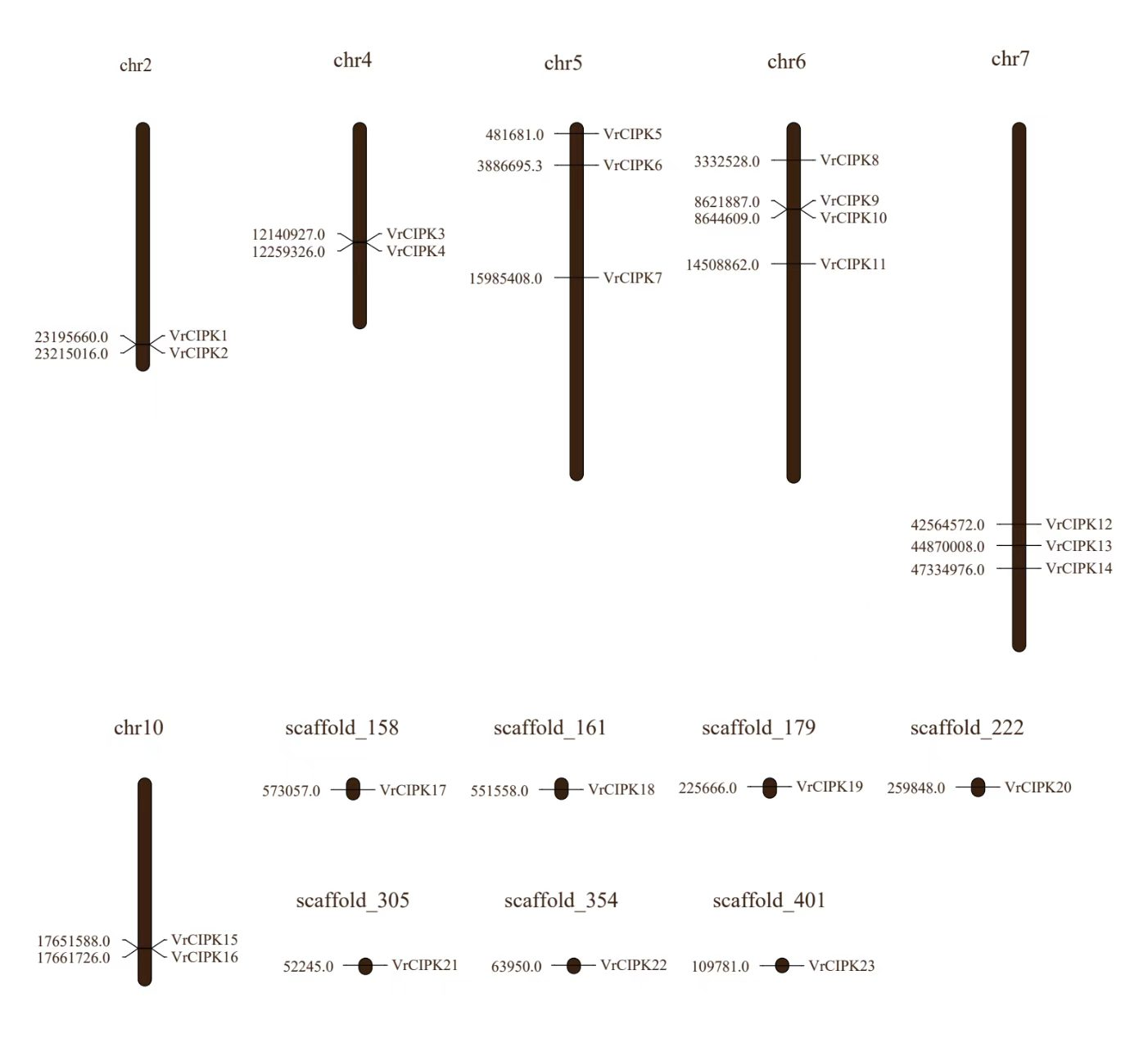

Supplement: Supplementary file 3 [file Image1.tif]

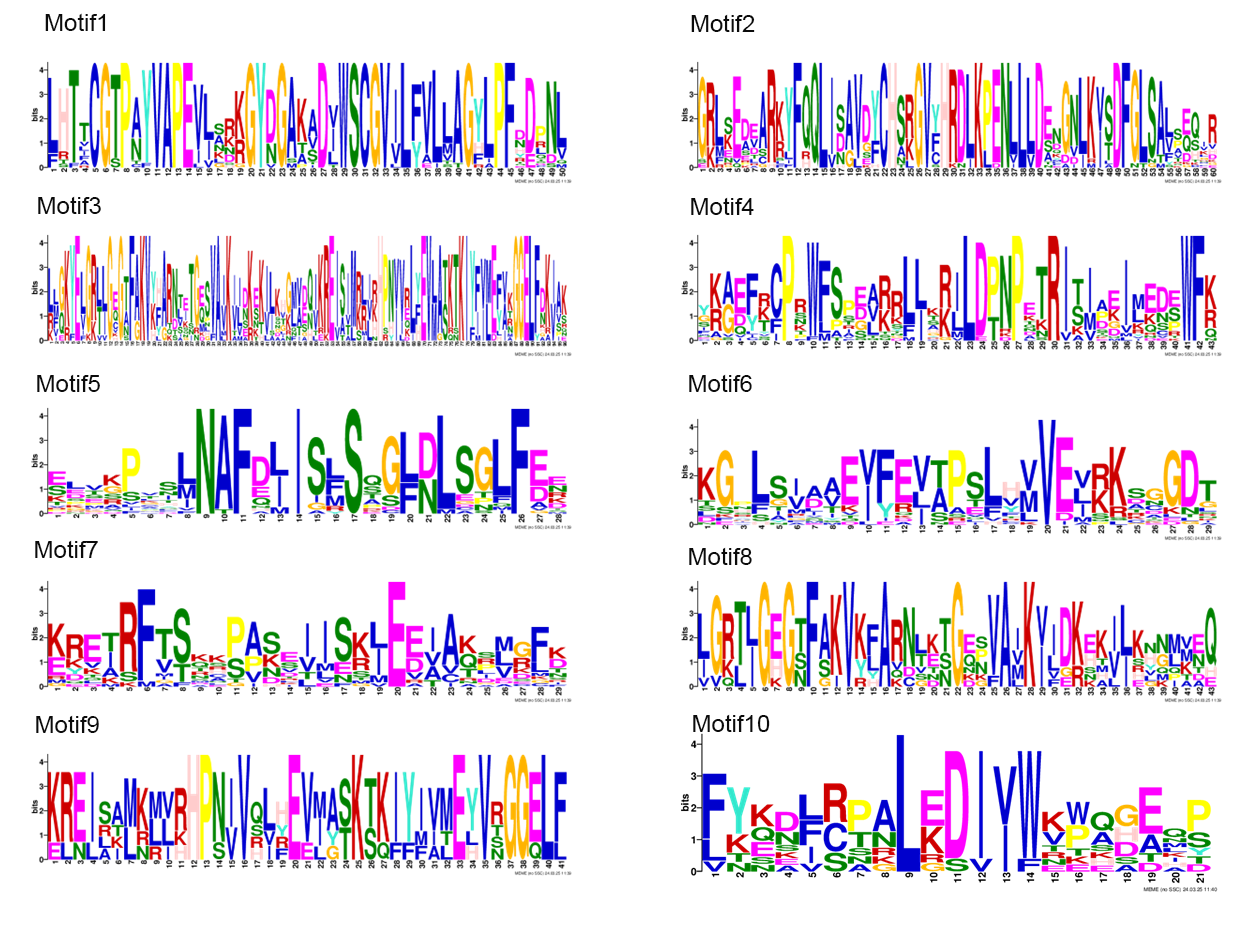

Supplement: Supplementary file 4 [file Image2.tif]

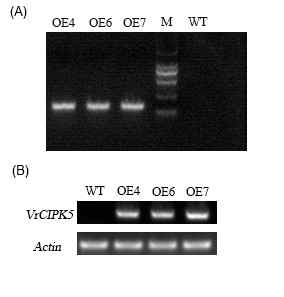

Supplement: Supplementary file 5 [file Image3.tif]
